# Improved microscale cultivation of *Pichia pastoris* for clonal screening

**DOI:** 10.1186/s40694-018-0053-6

**Published:** 2018-05-03

**Authors:** Alexander Eck, Matthias Schmidt, Stefanie Hamer, Anna Joelle Ruff, Jan Förster, Ulrich Schwaneberg, Lars M. Blank, Wolfgang Wiechert, Marco Oldiges

**Affiliations:** 1 Institute of Bio- and Geosciences, IBG-1: Biotechnology, Forschungszentrum Jülich GmbH, 52425 Jülich, Germany; 2 Institute of Biotechnology, RWTH Aachen University, 52074 Aachen, Germany; 3 iAMB - Institute of Applied Microbiology, ABBt – Aachen Biology and Biotechnology, RWTH Aachen University, 52074 Aachen, Germany; 4 Bioeconomy Science Center (BioSC), c/o Forschungszentrum Jülich GmbH, 52425 Jülich, Germany; 5 Computational Systems Biotechnology (AVT.CSB), RWTH Aachen University, 52074 Aachen, Germany

**Keywords:** *Pichia pastoris*, High throughput, Screening, Bioprocess development, Microbioreactor, Fed-batch, Phytase

## Abstract

**Background:**

Expanding the application of technical enzymes, e.g., in industry and agriculture, commands the acceleration and cost-reduction of bioprocess development. Microplates and shake flasks are massively employed during screenings and early phases of bioprocess development, although major drawbacks such as low oxygen transfer rates are well documented. In recent years, miniaturization and parallelization of stirred and shaken bioreactor concepts have led to the development of novel microbioreactor concepts. They combine high cultivation throughput with reproducibility and scalability, and represent promising tools for bioprocess development.

**Results:**

Parallelized microplate cultivation of the eukaryotic protein production host *Pichia pastoris* was applied effectively to support miniaturized phenotyping of clonal libraries in batch as well as fed-batch mode. By tailoring a chemically defined growth medium, we show that growth conditions are scalable from microliter to 0.8 L lab-scale bioreactor batch cultivation with different carbon sources. Thus, the set-up allows for a rapid physiological comparison and preselection of promising clones based on online data and simple offline analytics. This is exemplified by screening a clonal library of *P. pastoris* constitutively expressing AppA phytase from *Escherichia coli*. The protocol was further modified to establish carbon-limited conditions by employing enzymatic substrate-release to achieve screening conditions relevant for later protein production processes in fed-batch mode.

**Conclusion:**

The comparison of clonal rankings under batch and fed-batch-like conditions emphasizes the necessity to perform screenings under process-relevant conditions. Increased biomass and product concentrations achieved after fed-batch microscale cultivation facilitates the selection of top producers. By reducing the demand to conduct laborious and cost-intensive lab-scale bioreactor cultivations during process development, this study will contribute to an accelerated development of protein production processes.

**Electronic supplementary material:**

The online version of this article (10.1186/s40694-018-0053-6) contains supplementary material, which is available to authorized users.

## Background

The methylotrophic yeast *Pichia pastoris* is frequently used for the heterologous high-level expression of proteins for industrial and pharmaceutical applications [1, 2]. Advantages of this expression host are, among others, the availability of strong promotors enabling the heterologous production of proteins up to several grams per liter, growth to high cell densities in mineral medium in fed-batch cultivation, the possibility to target proteins for secretion and the low amount of secreted endogenous proteins [3].

As recently reviewed by Ahmad et al. [4], heterologous protein production in *P. pastoris* is affected by a number of biological and process parameters. For *P. pastoris*, the most important parameters are the selection of host strain, expression vector, promotor for target gene transcription, signal peptide for protein secretion, and clonal selection after transformation, which is necessitated by a pronounced variability, e.g., due to differences in the integration locus in the host genome, the gene copy number, or the orientation of multiple expression cassettes [5–11].

Further, protein production with *P. pastoris* is also affected by several process parameters, e.g., growth medium composition [12–14], carbon source selection [15, 16], temperature [17, 18], pH value [18, 19], and the dissolved oxygen concentration (DO) [20, 21].

Unfortunately, there is no straight forward way to combine these factors to achieve optimal production of different target proteins. With the advent of new tools for the generation of biological diversity (for a review see [22]), the bottleneck in bioprocess development is currently shifted from strain engineering to the evaluation of resulting strains. This creates a need for a repeated rapid testing of a large number of parameter combinations during bioprocess development, even if statistical tools for experimental design (DoE) are applied to reduce the experimental effort [23, 24].

High cultivation throughput for microbial screenings is currently achieved by the use of microtiter plates and shake flasks [25, 26], although drawbacks of these systems for aerobic cultivation are well documented [27, 28]. This can lead to results that are not reproducible in bioreactors and the false selection of production strains [19, 29]. On the other hand, bioreactor cultivation is laborious and expensive and its use is limited to later stages of process development [25, 26]. Parallel microbioreactor systems developed in recent years have evolved to promising alternative tools for screening and process development and have been successfully used for the cultivation of industrially important microorganisms [30, 31]. As reviewed earlier [27], especially the severe effect of oxygen limitation on cellular metabolism, resulting from low maximal oxygen transfer rates in microplates and shake flasks, may prevent the success of a screening experiment. To prevent oxygen limitation, low substrate and therefore low biomass concentrations are prevailing conditions during screening and represent striking differences to the conditions applied during protein production processes with *P. pastoris*. These are usually designed as carbon-limited fed-batch processes, in which the specific growth rate is determined by the applied feeding profile. The metabolic state of the cell at excess carbon conditions (e.g., carbon catabolite repression, overflow metabolism) clearly differs from carbon-limited conditions during fed-batch, as recently shown, e.g., on transcriptional and translational level [32]. In addition, only a limited insight into microbial physiology can be gained in screening experiments if growth is not monitored.

Taken together, insufficient data and inappropriate operational conditions can lead to the false selection of production strains in screening experiments [5, 25, 27]. Conditions in screening and early stages of process development should therefore already resemble those of the final production process [25, 33, 34]. Different mini- and microbioreactor systems have been developed in recent years to bridge this gap. Either shaken or stirred parallel vessels or plates, both allowing high oxygen transfer rates and mixing of the culture broth [26, 35], are used to perform parallel aerobic batch cultivation with culture volumes between < 1 and 100 mL [36]. The endowment with non-invasive optical monitoring allows the recording of important process parameters, biomass concentration and fluorescence as well as pH and DO with sensor spots. Thereby the informative content of parallel cultivation experiments is substantially increased.

Mini- and microscale cultivation have been applied for different microorganisms and results comparable to laboratory scale bioreactor cultivations have been reported in many cases [37–40]. Thus, such devices represent promising screening tools and can reduce the number of laborious and expensive bioreactor cultivations during process development [26, 30].

Considering both the potential of this technology and the importance of *P. pastoris* for protein production processes, surprisingly few application examples for microscale cultivation of this host have been published so far. Isett et al. established glycerol batch cultivation of *P. pastoris* in 4 mL medium in a 24 well plate miniature bioreactor system and showed successful control of temperature, DO and pH at increased cell densities after repeated manual substrate addition [41]. Using Applikon’s M24 miniscale cultivation device with controlled temperature, DO and pH, Holmes et al. [19] report a DoE approach to optimize methanol inducible (P_AOX1_) GFP production. In a further study, the influence of different feeding strategies on clone screening for inducible lipase production using P_AOX1_ was investigated in a RoboLector microscale cultivation platform and the obtained clone ranking could be confirmed in laboratory bioreactor cultivation [31, 42]. However, the realization of fed-batch processes in microscale cultivation systems is still in its infancy [31, 36, 43] and batch cultivation is prevailing for large clonal screenings [44].

We here adapt a BioLector microbioreactor system [39] for parallel cultivation of *P. pastoris*. 48-well baffled FlowerPlates used for cultivation ensure high oxygen transfer rates by high-frequency shaking. Online process parameters are recorded non-invasively by means of optical detection of scattered light as biomass-dependent signal and of online fluorescence for pH and DO using integrated optodes [39]. The aim of this study is to enable a facile and reliable screening of *P. pastoris* clonal libraries for protein production using the GAP-promotor. We therefore modified a high cell density cultivation medium for the parallel cultivation of *P. pastoris* in the BioLector system with different carbon sources and investigated scalability to laboratory bioreactor cultivation in terms of process characteristics like yields and growth rates. The application of this powerful set-up is applied to screen a *P. pastoris* library constitutively expressing *Escherichia coli* AppA phytase for the best production strain under different process conditions. Finally, fed-batch-like process conditions were realized in microscale cultivation to allow screening under process-relevant conditions, i.e. increased biomass and product concentrations.

## Results

### Adjustment of medium composition for microscale cultivation

**Fig. 1:**
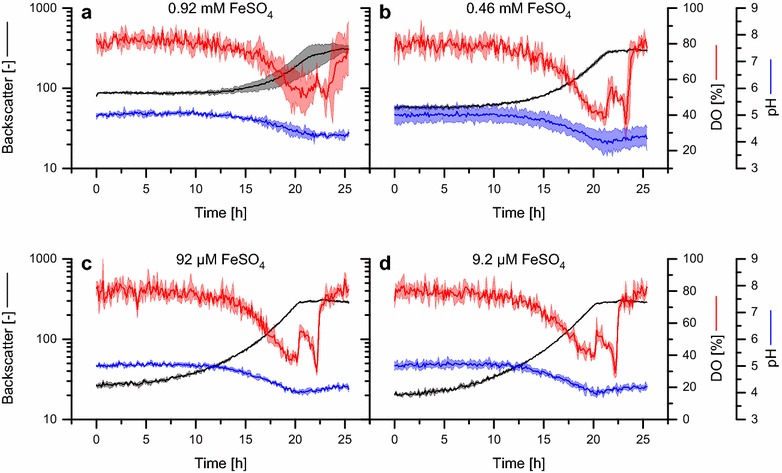
Reduction of the iron concentration in BSM prevents precipitation. The effect of reduced FeSO_4_ concentrations on medium stability and growth of *P. pastoris* in BSM was investigated in microscale cultivation (4% d-glucose, 150 mM PIPPS, pH 5.0, 0.8 mL, 1500 rpm, 30 °C). Different media were inoculated from the same pre-culture to an initial OD_600_ = 0.3 to ensure equal starting conditions. **a** 0.92 mM FeSO_4_ (original concentration); **b** 0.46 mM FeSO_4_; **c** 92 µM FeSO_4_; **d** 9.2 µM FeSO_4_. Light colors around solid lines show standard deviations from at least three individual wells

To accelerate bioprocess development for the eukaryotic protein production host *P. pastoris*, the aim of this study was to increase the cultivation throughput and to enable screening under process-relevant, defined conditions. Therefore, the BioLector microbioreactor system was adapted for miniaturized parallel cultivation of *P. pastoris*. In a first step, a basal salts medium (BSM) suitable for high cell density bioreactor cultivation was selected [45]. Customization of the medium for microscale cultivation was necessary since it does not contain a sufficient pH buffer, which is not required during bioreactor cultivation with controlled pH. Further, nitrogen is usually supplied partly by the use of ammonia, which is not possible in the BioLector. Therefore, 75.7 mM (NH_4_)_2_SO_4_ and 150 mM PIPPS pH 5.0 were added as nitrogen source and buffer, respectively. 4% d-glucose was used as carbon source. However, precipitation of medium components was observed at the standard cultivation pH 5.0, which caused a high background of the scattered light signal in the BioLector and thus interfered with online biomass detection (Fig. 1a). Therefore, further adjustment of the medium composition was necessary to meet the specific demands of BioLector microscale cultivation. Comparing the medium composition to growth media for other organisms, the iron concentration in BSM is remarkably high (0.92 mM FeSO_4_). In order to prevent the likely precipitation of iron salts in the medium, a reduction of the FeSO_4_ concentration and its influence on cellular growth was tested. When reducing the initial concentration to 0.46 mM (2-fold reduction), 92 µM (10-fold reduction), or 9.2 µM (100-fold reduction, Fig. 1b–d), the backscatter signal at the beginning of the cultivation, in case of the lowest concentration tested, decreased to a value similar to the backscatter of medium without FeSO_4_, thus showing that iron ions were the reason for precipitation which was avoided by a 100-fold reduction of FeSO_4_.

The online data for biomass, pH and DO (Fig. 1a–d) represent typical growth curves for batch cultivation of *P. pastoris*. After a lag phase, during which the backscatter, pH and DO remained constant for several hours, the increasing backscatter and the falling pH and DO reflect cellular growth. After the end of the growth phase, a second decrease of the DO signal occurred at t ~ 20–22 h, indicating the metabolization of by-products, which accumulated during batch cultivation. In accordance with a reduction in batch times, specific growth rates calculated from the backscatter signal (see methods) during the exponential growth phase slightly increased with the reduction of the FeSO_4_ concentration. While the specific growth rate was 0.18 ± 0.01 h^−1^ for 0.92 mM, it increased significantly (p < 0.05) to 0.22 ± 0.00 h^−1^ (0.46 mM), 0.26 ± 0.01 h^−1^ (92 µM) and 0.24 ± 0.01 h^−1^ (9.2 µM), respectively. The final backscatter was comparable for all conditions. Thus, the reduction of the FeSO_4_ content did not reduce the biomass yield and even increased the specific growth rate, showing sufficient supply of iron with no growth phenotype. A concentration of 9.2 µM FeSO_4_ in BSM (BSM_mod_) was used for all further cultivations.

**Fig. 2:**
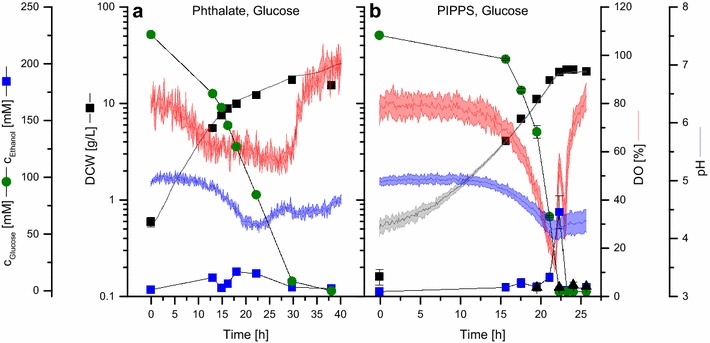
Comparison of buffers for microscale cultivation of *P. pastoris* at pH 5.0. **a** 100 mM potassium hydrogen phthalate pH 5.0 (1200 rpm, inoculated to OD_600_ = 1.0); **b** 150 mM PIPPS pH 5.0 (1500 rpm, inoculated to OD_600_ = 0.16). Cells were grown in 0.8 mL BSM_mod_ with 4% d-glucose at 30 °C. DCW concentrations were determined experimentally and calculated from online backscatter signals. Light colors around solid lines and error bars show standard deviations from at least three individual wells

A requirement for microscale cultivation in the BioLector is the addition of a buffer to the medium since the pH is not maintained otherwise. The choice of a buffer is crucial and can strongly influence the results of a cultivation experiment. We therefore selected two different buffers at pH 5.0. PIPPS was chosen because of its pK_a_ value (pK_a_ = 3.7). It is a tertiary amine sulfonic acid not showing weak organic acid characteristics and is thus assumed to be an inert buffer. Potassium hydrogen phthalate (pK_a,1_ = 5.4, pK_a,2_ = 3.0) is a common buffer for the cultivation of *P. pastoris* at low pH [45, 46]. Figure 2 shows growth curves for *P. pastoris* in BSM_mod_ containing 4% d-glucose and either 100 mM potassium hydrogen phthalate or 150 mM PIPPS at pH 5.0 in the BioLector. Online data for dry cell weight (DCW) calculated from the backscatter signal with the help of a calibration function (Additional file 1: Figure S1), pH, DO and experimental values for DCW, substrate and by-product concentrations are also shown. For the cultivation with potassium hydrogen phthalate a biphasic growth was observed. The specific growth rate dropped from 0.17 ± 0.0 to 0.06 ± 0.0 h^−1^ after 15 h.

In the presence of PIPPS the DCW values reveal exponential growth with a significantly increased (p < 0.05) specific rate of 0.23 ± 0.02 h^−1^. This is confirmed by the monotone decline of both the DO to a minimum of 14% and of the pH to 4.16 ± 0.16, which means no significant reduction compared to potassium hydrogen phthalate. Subsequently, in a second, short phase at t ~ 21–23 h the metabolization of ethanol, which was produced as by-product in the late exponential growth phase with maximal concentration of 70 ± 14 mM, was observed.

When comparing the results of cultivations with either PIPPS or potassium hydrogen phthalate as buffer, it is obvious that the latter exerted a negative effect on growth of *P. pastoris*. Therefore, all further cultivations in the BioLector were carried out using PIPPS.

### Scalability of microscale to laboratory bioreactor cultivation

**Fig. 3:**
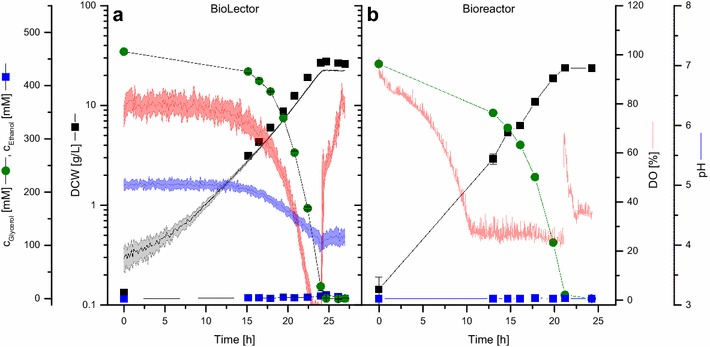
Scalability of BioLector and bioreactor cultivation of *P. pastoris* with glycerol as substrate. **a** BioLector cultivation with 4% glycerol in BSM_mod_ (150 mM PIPPS, pH 5.0, 0.8 mL, 1500 rpm, 30 °C, inoculated to OD_600_ = 0.3). Light colors around solid lines show standard deviations from at least three individual wells. **b** Bioreactor cultivation with 4% glycerol in BSM_mod_ (0.8 L, pH = 5.0 (NH_4_OH/H_2_SO_4_), DO = 30%, inoculated to OD_600_ = 0.6). Results from one representative bioreactor cultivation are shown (n = 3)

Cultivations in laboratory bioreactors were carried out to evaluate the scalability of the microscale cultivation set-up for *P. pastoris*. Cells were grown in a stirred tank reactor in 0.8 L BSM_mod_ with controlled pH (5.0) and DO (30%), respectively, representing an up-scale of the culture volume by a factor of thousand. Two different carbon sources, d-glucose and glycerol, were tested. Parameters for growth of *P. pastoris*::pGAPZαB*_appA* in the BioLector and the bioreactor are summarized in Table 1. Growth on 4% glycerol in the BioLector and bioreactor was exponential in both cases (Fig. 3). While the DO was controlled at 30% in the bioreactor, it steadily decreased during cultivation in the BioLector and was zero for the last 1.5 h. Nevertheless, no significant differences were measured for both the specific growth rate and the biomass yield, which were 0.23 ± 0.01 h^−1^ (bioreactor) and 0.24 ± 0.01 h^−1^ (BioLector), and 0.60 ± 0.02 g/g (both systems), respectively. No by-products were detected via HPLC-RI analysis in the culture supernatants in both cultivation setups.

**Table 1:** Specific growth rates and biomass yields for bioreactor and BioLector cultivations of *P. pastoris*

	d-Glucose		Glycerol	
	Bioreactor	BioLector	Bioreactor	BioLector
µ (h^−1^)	0.22 ± 0.01	0.23 ± 0.02	0.23 ± 0.01	0.24 ± 0.01
Y_x/s_ (g/g)	0.30 ± 0.02	0.48 ± 0.03	0.60 ± 0.02	0.60 ± 0.02

4% d-glucose or 4% glycerol were used as substrate in BSM_mod_ pH 5.0 (BioLector: 150 mM PIPPS, 0.8 mL, 1500 rpm, 30 °C; bioreactor: 0.8 L, pH = 5.0 (NH_4_OH/H_2_SO_4_), DO = 30%, 30 °C). Mean values ± standard deviations of at least 3 cultivations are shown

In contrast, the biomass yield for 4% d-glucose in the BioLector (0.48 ± 0.03 g/g, Table 1) was increased compared to the bioreactor cultivation (0.30 ± 0.02 g/g, Table 1), while the specific growth rate remained unchanged. The analysis of culture supernatants revealed differences in by-product accumulation with d-glucose as substrate between the two systems. In the BioLector, a transient maximal accumulation of 70 ± 14 mM ethanol was the only detectable by-product during aerobic growth (Fig. 2b), while the ethanol titer during bioreactor cultivation peaked at 141 ± 39 mM. In the latter case, the ethanol peak was followed by a transient acetate accumulation, which was then further metabolized (Additional file 1: Figure S2).

In conclusion, microscale cultivation of *P. pastoris* was successfully established using a modified high cell density growth medium. Reduction of the FeSO_4_ content was necessary to prevent precipitation of media components. PIPPS was chosen over potassium hydrogen phthalate for growth at pH 5.0. Growth parameters with glycerol as carbon source were in good agreement with results from laboratory-scale bioreactor cultivations, providing evidence that comparable results were achieved in both systems. However, cultivations with d-glucose point out important differences between both systems. After validation, the established microscale cultivation protocol for *P. pastoris* was applied to investigate the influence of clonal variability on protein production, both under batch and fed-batch-like conditions, and the results are described in the following section.

### Application of microscale cultivation for screening of clonal libraries under batch and fed-batch conditions

#### Initial selection of phytase secreting clones under batch conditions

Microscale cultivation in the BioLector was applied to screen a clonal library of *P. pastoris*::pGAPZαB*_appA* constitutively expressing and secreting AppA phytase from *E. coli* under control of P_GAP_. 47 individual clones resulting from the transformation of *P. pastoris* X-33 with the plasmid pGAPZαB*_appA*, previously linearized to facilitate chromosomal integration of the expression cassette, were cultivated in triplicate in the BioLector with 4% d-glucose as substrate to compare growth of individual clones and to identify the best phytase secreting ones.

Online backscatter signals for all clones were recorded during cultivation and converted to dry cell weight concentrations (Additional file 1: Figure S3 A). The resulting biomass data were used to calculate specific growth rates while biomass yields were determined experimentally at the end of the cultivation. Results for individual clones are shown in Additional file 1: Figures S4 and S5. Specific growth rates ranged from 0.22 to 0.26 h^−1^, while the mean biomass yield was 0.44 ± 0.02 g/g. A significant deviation (p < 0.05) of the specific growth rate from the parental strain was observed for 11 clones and for 9 clones with regard to the biomass yield, as determined by ANOVA and multiple comparison of means.

The online DO signal revealed two distinct phases during cultivation for all clones (Additional file 1: Figure S3 B), indicating transient accumulation of ethanol as a by-product in the presence of excess d-glucose. pH profiles for all clones were highly similar with the value decreasing steadily to a minimum of approximately 4.1 (Additional file 1: Figure S3 C).

**Fig. 4:**
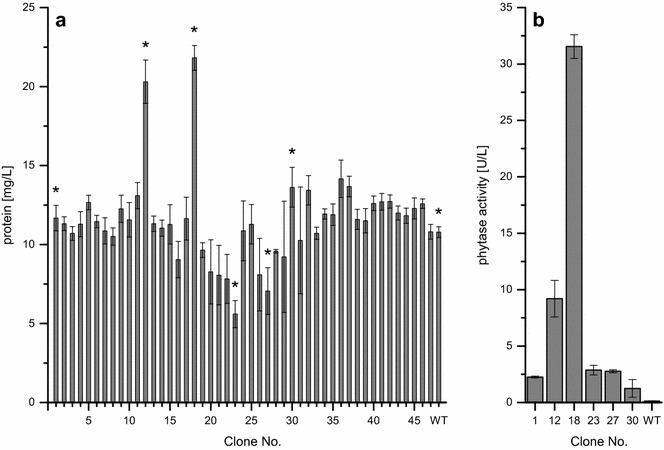
Clonal screening of a library of phytase secreting *P. pastoris*::pGAPZαB*_appA*. Clones were analyzed in microscale cultivation in BSM_mod_ with 4% d-glucose. **a** Protein concentrations in culture supernatants were determined using the Bradford assay. **b** For selected clones labeled with asterisks in a, phytase activities were determined after buffer exchange in a fluorescence assay using an artificial substrate. WT denotes the parental strain, *P. pastoris* X-33. Each clone was grown in triplicate, error bars show standard deviations

The purpose of this screening was the identification of transformants secreting phytase to the culture supernatant. Therefore, total protein concentrations in the supernatants collected after complete substrate and by-product consumption were initially used to identify protein secreting clones. On average, the supernatants of all tested transformants contained 11.5 ± 2.7 mg/L protein (Fig. 4a). As confirmed by ANOVA and multiple comparison of means, only clones 12 (20.3 ± 1.4 mg/L) and 18 (21.8 ± 0.8 mg/L) produced protein titers significantly (p < 0.05) exceeding the level of the parental strain (10.8 ± 0.3 mg/L). For these two as well as for clones 1, 23, 27, and 30 phytase activities were determined (Fig. 4b). The parental strain was used as negative control. Activities were highest for clone 18 (31.5 ± 1.0 U/L) and second highest for clone 12 (9.2 ± 1.6 U/L). Differences to the parental strain were found to be significant (p < 0.05) for all tested clones but clones 1, 23, 27 and 30 showed only very low phytase activities. Notably, values for protein concentrations in the supernatant do not fully correlate to phytase activities, since clone 12 showed protein concentrations similar to clone 18 but less than one third of its phytase activity.

This example points out the feasibility of screening of clonal libraries applying microscale cultivation of *P. pastoris*::pGAPZαB*_appA* in the BioLector. Best performing clones in terms of phytase secretion were identified and additional information on physiological parameters for individual clones, and qualitative information on substrate consumption and by-product formation was obtained.

In a next step, the microscale cultivation protocol was extended to enable clonal screenings under fed-batch-like conditions.

#### Adjusting carbon-limited conditions during microscale cultivation enables screening under process-relevant fed-batch conditions

Limiting the specific growth rate by controlled feeding of substrate allows taking control of metabolic rates during fed-batch cultivation.

**Fig. 5:**
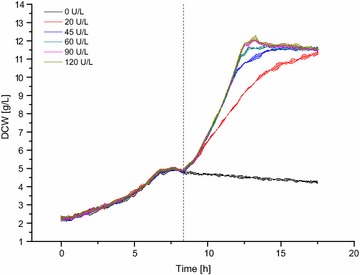
Establishing carbon-limited conditions during microscale cultivation of *P. pastoris* by application of enzymatic substrate release. 0.8 mL BSM_mod_ (150 mM PIPPS pH 5.0) with 0.5% glycerol as batch substrate and 1.8% dextrin was inoculated to OD 1.7 and incubated at 1300 rpm and 30 °C. Amyloglucosidase was added after 7 h (dashed vertical line). Dry cell weight concentrations were calculated from the online backscatter signal. Light colors around solid lines show standard deviations from three individual wells

We enabled fed-batch-like conditions for microscale cultivation of *P. pastoris* by the use of dextrin as a non-metabolizable glucose polymer and highly stable amyloglucosidase from *Aspergillus niger* for enzymatic glucose release. In a first experiment, different volumetric amyloglucosidase activities were tested to identify the boundaries for carbon-limited growth. BSM_mod_ was supplemented with 0.5% glycerol and 1.8% dextrin. After consumption of the batch substrate after 7 h (5 g/L DCW), 0–120 U/L amyloglucosidase was added to provide glucose monomers as additional carbon source at a constant rate (Fig. 5). A constant glucose release rate leads to linear growth and a decrease of the specific growth rate over time. After enzyme addition, increasing biomass concentrations were observed except for the negative control, confirming that *P. pastoris* does not thrive on dextrin as substrate. For high amyloglucosidase activities from 60 to 120 U/L (12–24 U/g DCW), growth curves are nearly identical and show exponential and thus non-limited growth. At 45 U/L (9 U/g DCW), growth started exponentially but became substrate-limited with increasing biomass concentration. In contrast, a linear increase of the DCW as expected for carbon limitation was observed after the addition of 20 U/L amyloglucosidase (4 U/g DCW). Thus, the specific amyloglucosidase activity of 4 U/g DCW was regarded as the upper limit of the specific glucose release rate in order to establish carbon-limited conditions in further cultivations.

Next, clones of *P. pastoris*::pGAPZαB*_appA* selected in batch screening (clones no. 1, 12, 18, 23, 27, 30, compare Fig. 4) were re-screened using the presented microscale fed-batch approach. Therefore, BSM_mod_ was supplemented with 2% glycerol and 10% dextrin as initial batch substrate and as substrate for enzymatic glucose release, respectively. After consumption of glycerol, 2.6 U/g DCW amyloglucosidase was added (25 U/L). Since this specific glucose release rate is below the defined upper limit of 4 U/g DCW, carbon limited growth conditions were ensured in the experiment. Addition of 25 U/L amyloglucosidase was repeated after 41.8 h (2.1 U/g DCW) and 66.0 h (1.8 U/g DCW) to re-adjust the glucose release rate in order to prevent a strong glucose limitation for the increasing biomass concentration.

**Fig. 6:**
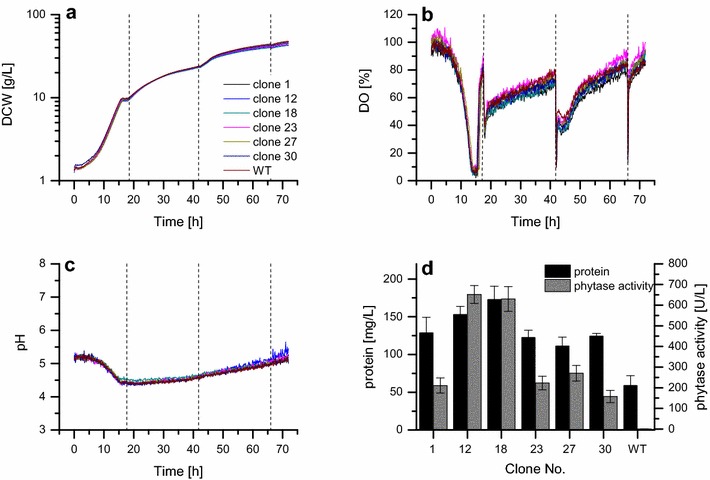
Microscale cultivation of phytase secreting *P. pastoris*::pGAPZαB*_appA* under carbon-limited conditions. Clones selected in a batch-screening (compare Fig. 5) were analyzed in microscale cultivation in 0.8 mL BSM_mod_ at pH 5.0 with 2% glycerol as batch substrate and 10% dextrin at 1300 rpm and 30 °C. 25 U/L amyloglucosidase was added after 17.7, 41.8, and 66.0 h (dashed vertical lines). **a** Dry cell weight concentrations calculated from the online backscatter signal; **b** Online measurement of dissolved oxygen concentrations; **c** Online measurement of pH; **d** Protein concentrations and phytase activities in culture supernatants. All values are corrected for evaporation. WT denotes the parental strain, *P. pastoris* X-33. Each clone was grown in quadruplicate, lines show mean values and error bars in **d** denote standard deviations

Time profiles of the online values for biomass, DO and pH (Fig. 6a–c) are nearly identical for all tested clones and the parental strain. Final dry cell weight concentrations were determined experimentally after 72 h and reached between 44.9 and 46.4 g/L. The exponentially increasing biomass concentrations indicate unlimited cell growth during the batch phase with a specific growth rate of 0.20 ± 0.00 h^−1^. At the same time, the DO decreased constantly and shortly dropped below 10% due to the high biomass concentrations at the end of the batch phase, before a DO peak reflected the consumption of the substrate. This oxygen limited phase could be avoided with lower batch substrate concentration. However, the likely short time by-product accumulation is expected to be rapidly consumed in the following carbon-limited phase. Then, the fed-batch phase was started by the addition of amyloglucosidase, resulting in a linear increase of the biomass signal over time. As expected, repeated addition of amyloglucosidase after 41.8 and 66.0 h lead to an accelerated but still limited growth due to the increase of the glucose release rate. Since the addition was performed offline under a sterile work bench, which took approximately 5 min, it caused short phases of oxygen limitation, but DO values recovered quickly. pH values decreased from 5.2 to 4.6 during batch cultivation but re-increased to > 5.0 at the end of the experiment.

The clonal ranking according to final protein titers and phytase activities (Fig. 6d) of the fed-batch screening confirmed batch results with one exception. In contrast to batch screening, protein titers and phytase activities were similar within the experimental error for clones 12 and 18 under substrate-limited conditions (153 ± 11 and 173 ± 18 mg/L, and 652 ± 43 and 630 ± 60 U/L). The background level of 59 ± 13 mg/L protein measured for the parental strain approximately corresponds to the amount of added amyloglucosidase, and no phytase activity was detected in this case. Clones 1, 23, 27, and 30 produced intermediate protein titers and phytase activities at similar levels (111 ± 12 to 128 ± 21 mg/L and 157 ± 30 to 270 ± 37 U/L, respectively). All culture supernatants were also analyzed via SDS-PAGE (Additional file 1: Figure S6). A protein band at 95 kDa visible after Coomassie staining represents amyloglucosidase, and one additional band is observed at 55 kDa. The native size of AppA phytase is 45 kDa, giving rise to the conclusion that AppA was secreted in non-native form and had been glycosylated by the posttranslational modification machinery in *P. pastoris* as observed earlier [47].

In conclusion, screening under batch- and substrate-limited conditions resulted in different clonal rankings since the performance of the second-best producer under batch conditions was identical to that of the best producer under carbon-limited conditions. Most strikingly, product titers were markedly increased after fed-batch cultivation. After correction for the background protein concentration of the parental strain the protein titer and phytase activity for clone 18 was increased 10-and 20-fold, respectively. Although batch-screening reliably distinguishes clones with low and increased protein secretion in shorter cultivation times, higher product titers produced during screening under carbon-limited conditions obviously facilitate the selection of best performing clones. The low increase of protein titers even for best phytase producers compared to the background level caused by the secretion of host proteins (parental strain) in batch cultivation presents a possible source of error. Therefore, screening under fed-batch conditions has the advantage of resulting in more reliable and process-relevant results, especially for proteins that are produced at low levels in *P. pastoris* only.

## Discussion

In this study, we applied the 48 well plate based BioLector microbioreactor system [39] for the cultivation of *P. pastoris*, which is an important protein production host. By tailoring a defined high cell density medium for batch and carbon-limited parallel growth experiments, we show that process-relevant and scalable screening conditions can be achieved. Although simple in its application, successfully setting up microscale BioLector cultivation for *P. pastoris* required to consider several aspects. The selection of a suitable pH buffer is a crucial requirement since the BioLector does not allow pH control. *P. pastoris* grows at a broad pH range between 3 and 7 [48], but changes in pH alter the specific growth rate, can activate host proteases [49], and were shown to affect GFP production [19]. We thus compared growth at pH 5.0 using two different buffers, PIPPS and potassium hydrogen phthalate. It was obvious that phthalate severely inhibits growth. This was probably attributed to an uncoupling of the proton motive force due to its weak organic acid properties combined with aromatic carbon backbone, facilitating membrane diffusion. Additionally, a biochemical inhibition of glycolytic enzymes from *Saccharomyces cerevisiae* by phthalic acid has been noted earlier in vitro [50]. In contrast, no growth inhibition after the addition of PIPPS was observed.

Precipitation of media components is a frequent drawback of high cell density growth media. This has been recognized as a problem potentially leading to nutrient starvation and interfering with optical process analyses [26, 51]. Reliable process information via optical online detection of biomass, pH, and DO in the BioLector was mandatory for the success of this study, which made stabilizing the medium by adjusting its composition a prime task. It was found that reducing the original FeSO_4_ concentration (0.92 mM) by a factor of 100 prevented precipitation (Fig. 1). High iron ion concentrations above 1 mM are also found in other *Pichia* media described in literature [52, 53] and possibly are needed to support growth to very high biomass concentrations in fed-batch cultivation. In a study on medium optimization, Isidro et al. report that iron ions inhibit metabolic activity of *P. pastoris* even after 10-fold reduction to 0.1 mM [54]. In our experiments, a slight increase of the specific growth rate was measured upon FeSO_4_ reduction to 9.2 µM. Further, the biomass yield was not altered up to the maximum tested substrate concentration of 4%, thus showing that cells did not face iron limitation at reduced FeSO_4_ concentrations.

When testing the scalability of physiological parameters for *P. pastoris* between BioLector and laboratory scale bioreactor cultivations, specific growth rates and biomass yields were in good agreement for glycerol. Surprisingly, growth yields on d-glucose were lower for bioreactor cultivations. This effect was observed reproducibly in biological triplicate bioreactor cultivation. Main differences between the two cultivation systems are the absence of pH and DO control in the BioLector as well as in aeration. As a consequence of the high and constant oxygen transfer rate at the adjusted setting of the device (110 mM/h as specified by the manufacturer), cells were exposed to high DO levels in the BioLector for the major part of the cultivation. According to earlier observations [15], the lower and constant DO setpoint of 30% in the bioreactor probably accounted for the formation of ethanol and acetate as by-products and the reduced biomass yield, pointing towards the necessity of increased DO levels to fully saturate the respiratory chain under the applied conditions. Further, oxygen is supplied by surface aeration in the BioLector while the medium is supplied with a constant air stream in the bioreactor. In the latter case, this might lead to an increased loss of volatile by-products like ethanol via evaporation and might thereby prevent its re-assimilation. We also observed a reduced by-product accumulation during bioreactor cultivation at lower constant pH values of 3 and 4 (own unpublished results), indicating a combined effect of both DO and pH on the physiology of *P. pastoris*.

The presented microscale cultivation set-up was applied to screen a library of *P. pastoris*::pGAPZαB*_appA* transformants secreting AppA phytase from *E. coli*.

By screening of 47 *P. pastoris*::pGAPZαB*_appA* transformants in BSM_mod_ with 4% d-glucose in microscale cultivation, significantly increased protein concentrations and phytase activities in the supernatant compared to the parental strain were measured for two clones. Protein titers still were only low, which might be due to the fact that we did not include steps for the selection of multicopy clones during strain construction. Clones carrying multiple copies of the expression cassette often show higher expression levels than those with only one or few copies [55]. This correlation not always holds true, especially in the case of secreted proteins, where the secretion apparatus may become a bottleneck in protein production [10, 56].

Specific growth rates and biomass yields were very similar. Nevertheless, it is common knowledge that protein overproduction can lead to reduced biomass yields and growth rates [57, 58]. The possibility for parallel phenotyping with the help of online process data already during screenings makes it possible to select those that promise to perform well after scale-up to bioreactor cultivation. Non-invasively monitoring DO and pH during screening further assures reproducible conditions and is an advantage compared to cultivation in standard microplates.

Some solutions to transfer carbon-limited fed-batch cultivation to microscale have been established in the past as recently reviewed [59]. For example, repeated dosing of a substrate bolus to the culture has been used to increase biomass and product concentrations in microscale cultivation [31]. However, this approach cannot be regarded as a true fed-batch process because it leads to alternating conditions of excess substrate (maximum specific growth rate) and substrate limitation (zero growth). Continuous feeding of small volumes required for microscale fed-batch cultivation is currently still limited by the availability of suitable micropumps, although first prototypic examples have been described [43, 59, 60]. This problem has been circumvented by providing substrate via diffusion [61, 62]. Another possibility is the enzymatic substrate release from oligo- or polymeric inert compounds like starch [31, 60, 63] or sucrose [43] at a constant rate. Contrary to exponential substrate feeding, which can be applied during lab-scale bioreactor cultivation to adjust a constant specific growth rate, a constant substrate release rate will lead to a decreasing specific growth rate over time. Since an interdependence of specific growth rate and protein production rate is well documented [15, 64, 65], the development of more flexible feeding strategies suitable for microscale cultivation remains an important issue for further optimization.

A thorough characterization of cultivation profiles in batch-mode was performed in this study to enable a direct comparison of results from microscale and laboratory scale to ensure scalability of the developed cultivation setup before advancing to the development of a microscale fed-batch cultivation protocol. For the latter, we employed enzymatic substrate release, since it is easily applicable and well suited to infer carbon-limited conditions during microscale cultivation. When screening a clonal library of phytase producing *P. pastoris* under carbon-limited conditions, biomass concentrations, protein titers and phytase activities in the supernatant were strongly increased compared to batch cultivation. In batch screening, it was possible to identify clones with increased phytase production in a shorter time, but the clonal ranking for fed-batch-like screening was different concerning the two best producers. Even more drastic changes in clonal rankings for different operational modes were reported by Scheidle et al. for GFP production in *Hansenula polymorpha* [33]. Further, clones with a reduced maximum growth rate, e.g., due to a metabolic burden caused by protein overproduction, might be deselected after batch screening, although they could be well suited for protein production in a carbon-limited process Taken together, the possibility to perform clonal selections under fed-batch conditions clearly presents a major advantage over screening in batch mode.

Application of the microscale cultivation protocol could comprise not only screening, but also the rapid initial testing of process parameters during process optimization. Variables like C- and N-source, media composition, temperature and initial pH could be tested, e.g., as part of DoE studies, to reduce the number of bioreactor cultivations. Further, the developed microscale cultivation set-up for *P. pastoris* also allows an easy integration into liquid handling stations [31, 42]. Miniaturization and parallelization of microbial cultivation will eventually contribute to an acceleration of bioprocess development.

## Conclusions

In this study, we developed, validated and applied a setup for the screening of clonal libraries of protein producing *P. pastoris* employing the constitutive GAP promotor in a parallel microbioreactor system. First, a defined high cell density cultivation medium was modified by the addition of N-source, the selection of an inert buffer for cultivation at pH 5 and the FeSO_4_ concentration was reduced to prevent precipitation. Scalability of cultivation results from microscale to laboratory bioreactor cultivation was ensured by the comparison of physiological parameters (Y_X/S_, µ) for growth on d-glucose and glycerol as substrates in both scales and for d-glucose even an improvement of Y_X/S_ was found in microscale. In addition to laborious offline measurements, online growth signals provided by the cultivation system were used for the parallel determination of physiological parameters, e.g., the calculation of specific growth rates from online backscatter measurements, and online monitoring of DO and pH ensured reproducible conditions throughout the conducted screening experiments.

Enzymatic substrate release was adopted using a glucose polymer and glucose liberating amyloglucosidase to impose carbon-limited growth conditions, allowing screening under process relevant fed-batch conditions in microscale cultivation. Therefore, different glucose release rates were adjusted by the addition of amyloglucosidase and suitable cultivation conditions could be identified.

The established microscale cultivation was applied for the screening of a clonal library of *P. pastoris*::pGAPZαB*_appA* constitutively secreting AppA phytase from *E. coli* both in batch and fed-batch mode. Although the former allowed a rapid selection of clones producing increased extracellular protein titers and phytase activities, protein concentrations and phytase activities were increased 10- and 20-fold, respectively, in fed-batch microscale cultivation. DCW concentrations > 45 g/L were reached at fully aerobic conditions, exceeding maximum DCW concentrations during batch cultivation more than 2-fold. Most importantly, differences in the clonal ranking were found between the two operational modes, i.e. batch and fed-batch. This confirms the necessity of reliable advanced screening technologies such as is presented in this study to accelerate the future development of bioprocesses.

## Methods

### Chemicals and growth media

All chemicals and enzymes were obtained from Sigma Aldrich (Steinheim, Germany) or Carl Roth (Karlsruhe, Germany) if not mentioned otherwise and were of analytical grade. PIPPS was purchased from Merck (Darmstadt, Germany).

YPD complex medium consisted of 10 g/L yeast extract, 20 g/L peptone and 20 g/L d-glucose. For solid YPD medium, 16 g/L agar–agar was added. Basal salts medium (BSM) [45] was used with modifications, consisting of 81.1 mM ortho-phosphoric acid, 9.4 mM MgSO_4_ × 7 H_2_O, 1 mM CaSO_4_ × 2 H_2_O, 16.4 mM K_2_SO_4_, and 75.7 mM (NH_4_)_2_SO_4_. The pH was adjusted to 5.0 with KOH. A suitable buffer was added for cultivations without pH control (see results). After sterilization, 4 mL/L trace salts solution was added containing 24 mM CuSO_4_ × 5 H_2_O, 0.53 mM NaI, 19.87 mM MnSO_4_ × H_2_O, 0.83 mM Na_2_MoO_4_ × 2 H_2_O, 0.32 mM H_3_BO_4_, 2.1 mM CoCl_2_ × 6 H_2_O, 150 mM ZnCl_2_, 230 mM FeSO_4_ × 7 H_2_O and 0.82 mM biotin. For BSM_mod_, the concentration of FeSO_4_ × 7 H_2_O in the trace salts solution was reduced to 2.30 mM.

### Strain construction

*Escherichia coli* DH5α [66] was used for general cloning, whereas *P. pastoris* X-33 (Invitrogen, Carlsbad, USA) was used for gene expression. The gene *appA* (gene ID 946206) from *E. coli* K-12 substrain MG1655 was codon optimized for *P. pastoris* and synthesized by GeneArt™ (Regensburg, Germany) and inserted, after digestion with *Pst*I and *Not*I, into the vector pGAPZαB (Invitrogen). The resulting plasmid pGAPZαB_*appA* was transformed into *P. pastoris* X-33 by electroporation using the method described by Wu and Letchworth [67], resulting in the strain *P. pastoris*::pGAPZαB*_appA*.

### Cultivation of *P. pastoris*

Strains were preserved as cryocultures in 30% glycerol at − 80 °C. For cultivation of *P. pastoris* strains, cells were plated on YPD agar containing 100 µg/mL Zeocin (Invivogen, Toulouse, France) if required and incubated at 30 °C for 2 days. Then, 10 mL YPD liquid medium were inoculated and incubated in a rotary shaker (Multitron Standard, Infors HT, Einsbach, Germany) at 250 rpm and 30 °C for 8 h. A second pre-culture in 10–100 mL basal salts medium supplemented with 150 mM PIPPS (pH 5.0) and 1% of the same carbon source as in the main culture was inoculated and cultivated under the same conditions for 16 h. Pre-cultures were grown in baffled shake flasks ten times the volume of the culture broth.

Microscale cultivation of *P. pastoris* in a BioLector (m2p-labs, Baesweiler, Germany) was performed using MTP-48-BOHL FlowerPlates, which are equipped with pre-calibrated optodes for online DO (dynamic range 0–100% saturation) and pH (dynamic range pH 2.25–5.75) measurement (m2p-labs). The plates were sealed with gas-permeable F-GP-10 sealing foils (m2p-labs). Cultivation settings were 30 °C, 1500 rpm, and atmospheric humidity control ≥ 85%. 800 µL BSM or BSM_mod_ were supplemented with 150 mM PIPPS pH 5.0 and 4% of either d-glucose or glycerol. For cultivation under carbon-limited conditions, 10% dextrin from potato starch and amyloglucosidase from *A. niger* (see “Results”) were added. Media were inoculated from a pre-culture to an initial OD_600_ between 0.1 and 1.7. Growth was monitored online by backscatter measurement (620 nm, gain = 15).

Bioreactor cultivations were carried out in 0.8 L BSM_mod_ with 75.7 mM (NH_4_)_2_SO_4_ without buffer at 30 °C in DASGIP Bioblock stirred tank reactors (DASGIP, Jülich, Germany) equipped with electrodes for pH and DO and off-gas analysis for O_2_ and CO_2_. The pH was maintained at 5.0 by addition of 18% (v/v) NH_4_OH and 8% (v/v) H_2_SO_4_. The DO was controlled by applying a stirrer cascade (400–1200 rpm), while the airflow was constant at 60 L/h sterile ambient air (0.2 µm PTFE filter, DIAFIL, Wieliczka, Poland).

### Analytical procedures

DCW concentrations were determined gravimetrically. 0.5–2 mL sample were collected in dried Eppendorf tubes (2 days at 80 °C, 1 day in a desiccator) and centrifuged for 5 min at 13,000 rpm (Biofuge pico, Heraeus, Hanau, Germany). The pellet was washed in 0.9% NaCl solution and dried as described above.

Protein concentrations in culture supernatants were determined using Bradford reagent (Sigma Aldrich) according to the manufacturer’s instructions with bovine serum albumin as standard. SDS-PAGE for analysis of culture supernatants was performed using 12% TruPAGE™ (Sigma Aldrich) polyacrylamide gels and Coomassie staining according to the manufacturer’s instructions.

A fluorescence assay was applied to determine phytase activities in culture supernatants using 4-MUP as substrate [68]. Proteins excreted to the culture supernatant were separated from low molecular weight compounds via ultrafiltration (Nanosep Omega 10 kDa, Pall, Dreieich, Germany) using 3 volumes of 50 mM sodium acetate (pH 5.0) for washing and as exchange buffer. 50 µL of the resulting sample was mixed with 50 µL 1 mM 4-MUP in 50 mM sodium acetate buffer (pH 5.0) in a transparent 96 well plate and the increase of fluorescence (excitation 336 nm, emission 448 nm, 25 °C) was recorded for 15 min in an Enspire microplate reader (Perkin Elmer, Rodgau, Germany). Enzyme activities were calculated from the resulting slope using a calibration curve of the reaction product.

Sugars and organic acids in the culture supernatant were analyzed on an Agilent 1100 series HPLC system equipped with RI and DAD detector (Agilent Technologies, Waldbronn, Germany). As stationary phase, an organic acid resin (300 × 8 mm, CS-Chromatographie Service, Langerwehe, Germany) was used at 80 °C and isocratic elution with 0.6 mL/min 100 mM H_2_SO_4_. Samples were analyzed quantitatively by external calibration.

### Calculation of physiological parameters and rates

Biomass yields were calculated from final DCW concentrations and initial substrate concentrations. For the calculation of specific growth rates from the backscatter measurement in the BioLector, the signal was converted to DCW concentrations with the help of a non-linear calibration (Additional file 1: Figure S1) and by fitting an exponential growth model to the progression of the DCW over time. For the calculation of all rates and yield coefficients for BioLector cultivations, the culture volumes were corrected for 9.09% evaporation per day, which was determined gravimetrically and assumed to be linear over time.

### Statistical analysis

ANOVA and multiple comparison of means tests were performed with Matlab R2016b (The MathWorks GmbH, Ismaning, Germany).

## Additional file

**Additional file 1.** Supplemental material.

## Abbreviations

BSM: basal salts medium
BSM_mod_: basal salts medium, modified
DCW: dry cell weight
DO: dissolved oxygen
HPLC: high pressure liquid chromatography
µ: specific growth rate
PIPPS: piperazine-*N*,*N*′-*bis*(3-propanesulfonic acid)
rpm: rounds per minute
Y_x/s_: biomass yield on substrate
