# Supplementary material for: Improved microscale cultivation of Pichia pastoris for clonal screening
Source: Fungal Biol Biotechnol. 2018 May 3;5:8. doi: 10.1186/s40694-018-0053-6 (PMC5932850; doi:10.1186/s40694-018-0053-6)
Supplement: Supplementary file 1 — Additional file 1. Supplemental material. [file 40694_2018_53_MOESM1_ESM.pdf]

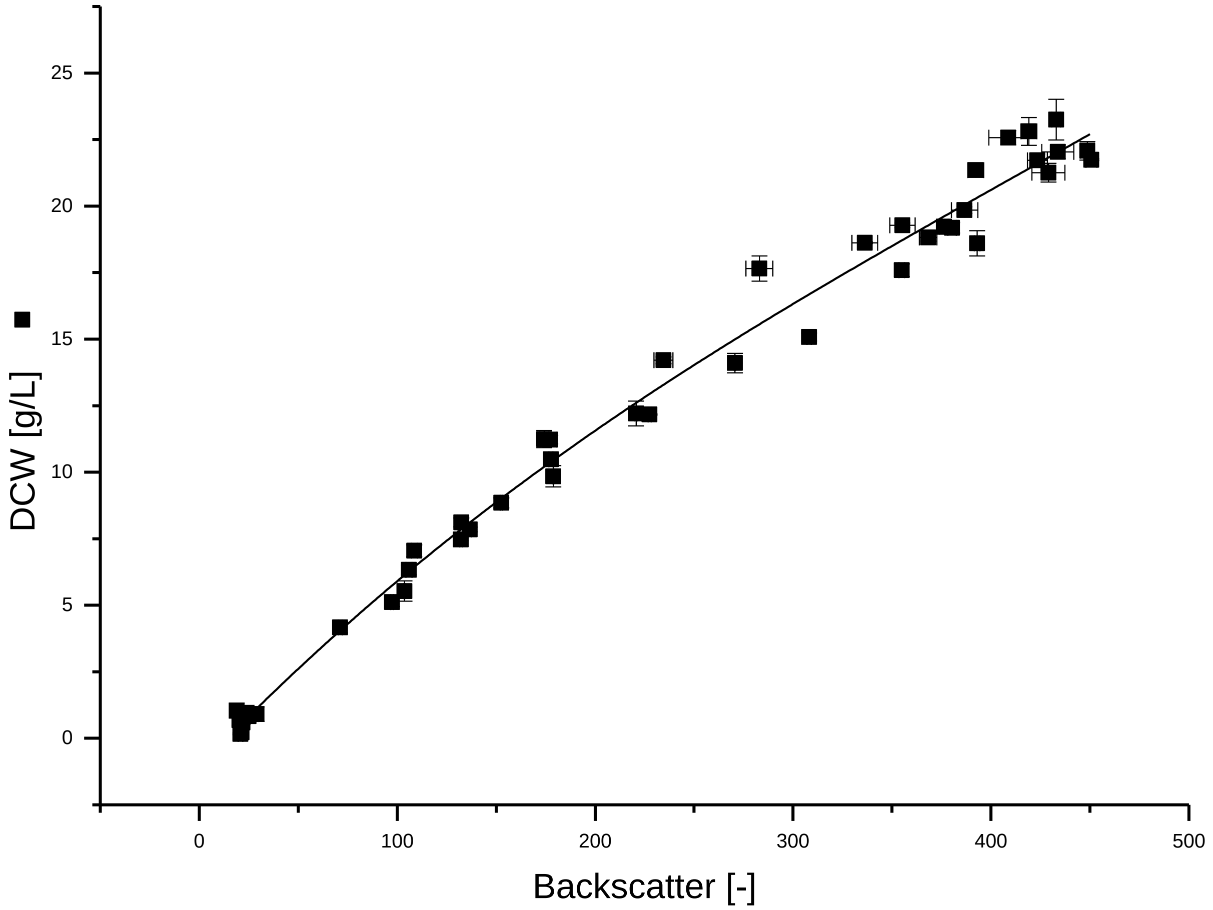

Figure S1: Correlation of dry cell weight and BioLector online backscatter signal. DCW concentrations of samples from five microscale cultivation runs in BSM_{mod} (4% D-glucose, 150 mM PIPPS, pH 5.0, 0.8 mL, 1500 rpm, 30°C) were plotted against online backscatter values (620 nm, gain 15) and fitted to a third degree polynomial (solid line, R^2 = 0.99). DCW and backscatter were determined as triplicates, error bars show standard deviations.

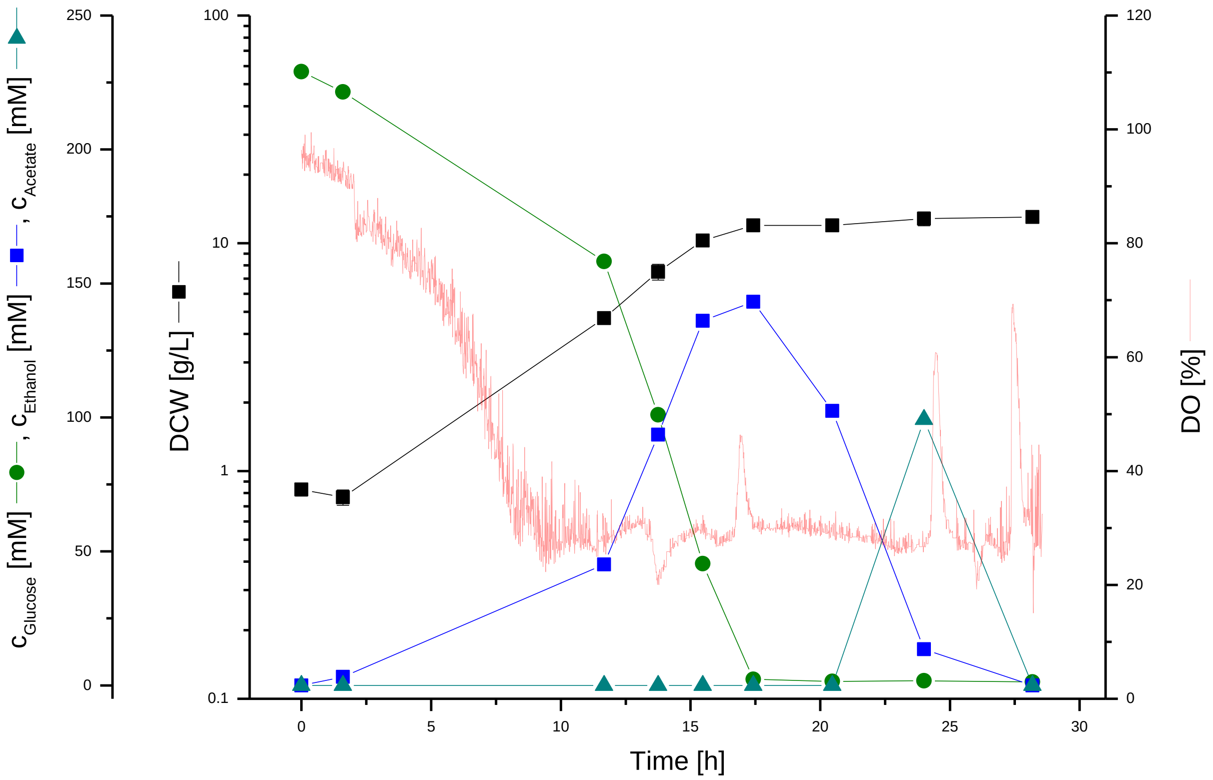

Figure S2: Bioreactor cultivation of *P. pastoris* on D-glucose. Cultivations were performed in BSM_{mod} (4% D-glucose, 0.8 L, pH = 5.0 (NH_4OH / H_2SO_4), DO = 30%, inoculated to OD_{600} = 1.0). Results from one representative out of three cultivations are shown.

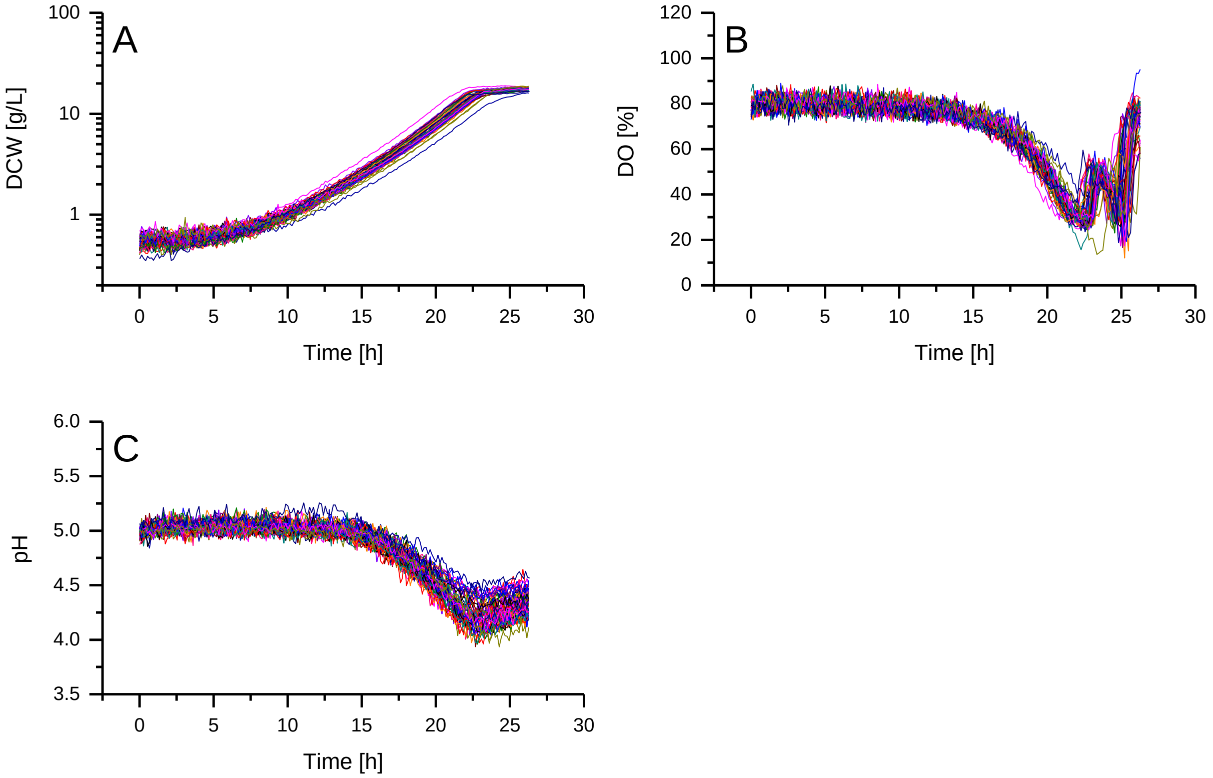

Figure S3: Online growth data for parallel cultivation of 47 transformants of AppA phytase secreting *P. pastoris*::pGAPZαB_*appA*. DCW concentrations (A), DO (B) and pH (C) are shown for a clonal screening in BSM_{mod} plus 4% D-glucose (150 mM PIPPS, pH 5.0, 0.8 mL, 1500 rpm, 30°C). DCW concentrations were calculated from online backscatter measurements. Each clone was cultivated in triplicate, lines show mean values.

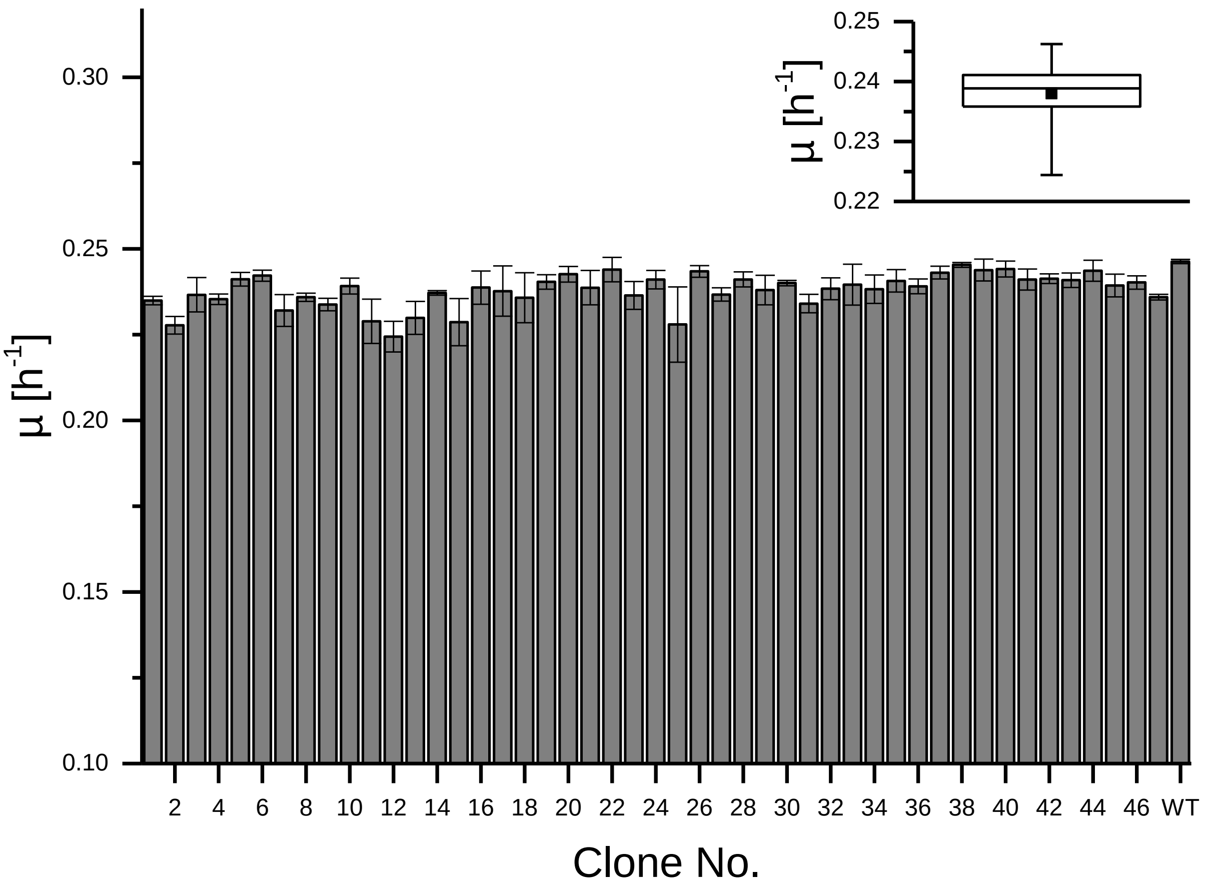

Figure S4: Specific growth rates for 47 transformants from a clonal library of AppA phytase secreting *P. pastoris*::pGAPZαB_*appA*. Values were calculated from the online biomass signal with the help of a non-linear correlation (figure S1). Each clone was cultivated in triplicate, error bars show standard deviations. Boxes show the 25 – 75 percentile, median (line) and mean value (square). Whiskers represent minimum and maximum values.

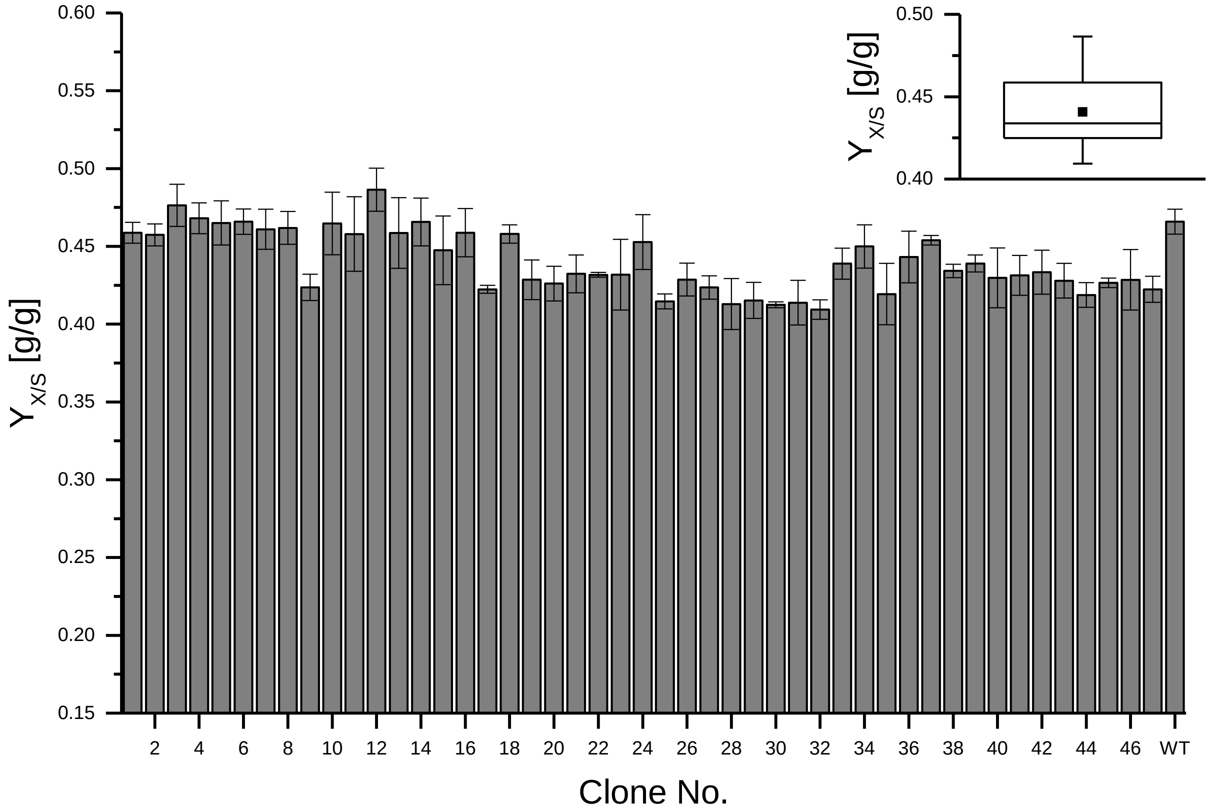

Figure S5: Biomass yields for 47 transformants from a clonal library of AppA phytase secreting *P. pastoris*::pGAPZαB_*appA*. Biomass yields ($Y_{X/S}$) were calculated from DCW concentrations determined experimentally at the end of the cultivation. Each clone was cultivated in triplicate, error bars show standard deviations. Boxes show the 25 – 75 percentile, median (line) and mean value (square). Whiskers represent minimum and maximum values.

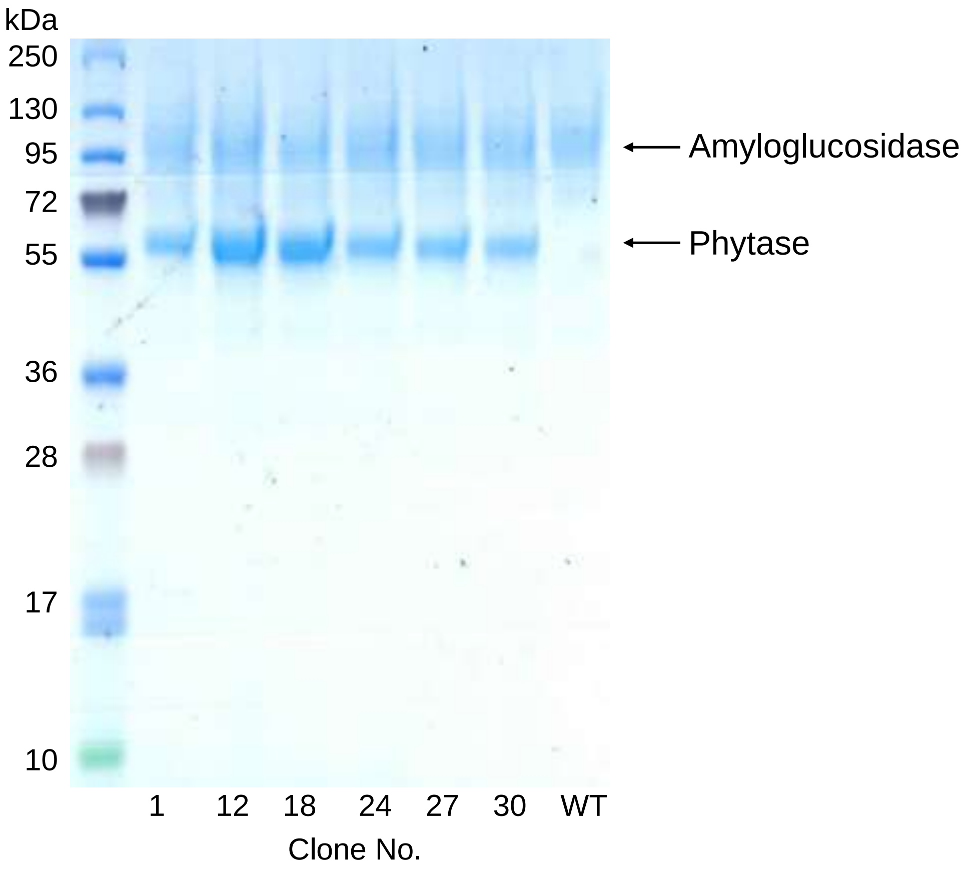

Figure S6: SDS-PAGE analysis of phytase production during microscale cultivation under carbon-limited conditions. Cultivation was performed in 0.8 mL BSM_{mod} at pH 5.0 with 2% glycerol as batch substrate and 10% dextrin (1300 rpm and 30°C). At three time points, 25 U/L amyloglucosidase was added. For all clones 20 µL of culture supernatant sampled after 72 h were analyzed.
